# The Origin Link Between “Śląski” Cheese and the Silesia Region: A Basis for Obtaining Protection for Geographical Indications

**DOI:** 10.3390/foods14030338

**Published:** 2025-01-21

**Authors:** Sylwia Chudy, Jakub Kępiński, Agnieszka Makowska

**Affiliations:** 1 Department of Dairy and Process Engineering, Faculty of Food Science and Nutrition, Poznań University of Life Sciences, 60-637 Poznań, Poland; 2 Poznań Chair of Civil, Commercial and Insurance Law, Faculty of Law and Administration, Adam Mickiewicz University, 61-712 Poznań, Poland; jakub.kepinski@amu.edu.pl; 3 Department of Food Technology of Plant Origin, Faculty of Food Science and Nutrition, Poznań University of Life Sciences, 60-637 Poznań, Poland; agnieszka.makowska@up.poznan.pl

**Keywords:** “harceński” cheese, “śląski” cheese, Silesia region, Protected Designation of Origin, protected geographical indications

## Abstract

This paper presents the history of cheese produced in the Silesian region (Poland). The purpose of the research was to collect documentation on cheese, for which the name “śląski” cheese (“Silesian” cheese) is adopted in this paper. The research method was a query, and the research materials were archival documents collected in Polish libraries and archives. Conducting a comprehensive search made it possible to collect relevant information on “śląski” cheese. The authors also carried out a survey to obtain data on consumer behaviour in the regional food market. Based on the research, there is a strong rationale for taking steps to safeguard the name “śląski” cheese by acquiring a Protected Designation of Origin or Protected Geographical Indication status. As a significant cheese producer in the European Union, Poland should promote cheeses associated with a specific region. “Śląski” cheese has the potential to become a symbol of the Silesia region in the southwestern part of Poland. This research is an example of documentation gathering and could be helpful to all those who are trying to obtain geographical indications for regional products.

## 1. Introduction

The historical documentation of food products plays a key role in understanding both culture and the evolution of human eating habits. The significance of this practice extends well beyond the mere documentation of culinary formulas or technological methodologies.

This article focuses on one regional product—“śląski” cheese (“Silesian” cheese). This cheese was produced for many years in a southern part of Poland called Silesia. The authors aimed to establish an “origin link” between this cheese and its geographical origin. By highlighting its regional roots, this cheese can potentially serve as a significant element for promoting the region in the future.

One of the key aspects of historical documentation is the preservation of cultural heritage. The importance to preserving the cultural heritage was expressed, inter alia, in the UNESCO Convention for the Safeguarding of the Intangible Cultural Heritage (ICH), adopted in 2003 [1]. However, according to Ubertazzi, “Convention lacks rules on safeguarding ICH from transnational misappropriation. In the absence of these rules, States Parties to the Convention have adopted measures to safeguard ICH across borders, such as intellectual property rights (IPRs), in particular protected geographical indications” [2]. Thus, GIs can be one way of protecting cultural heritage [3].

Cuisine is an integral part of national and regional identity. The recipes, culinary techniques, and ingredients used to produce food in a region reflect its history, geographical, and social conditions. Documenting these elements helps to preserve knowledge of the past, which could be forgotten as a result of globalisation and cultural homogenisation. The historical documentation of food products is a valuable resource for researchers. It allows for the analysis of how various socio-economic factors influence diets and how eating habits and cooking techniques have changed over time. This provides a better understanding of how people in the past coped with the challenges of food production, storage, and preparation. The evolution of food products is closely linked to the history of agriculture and trade. Documentation can help to trace how different plants and animals have been used in agriculture and how agricultural techniques have changed over the centuries. It can also show how trade influenced the availability and popularity of different foods [4,5].

In the current era of rapid technological and lifestyle changes, a large number of traditional dishes and techniques of food preparation could be forgotten. Keeping records should help to reintroduce, promote, and pass them on to future generations [6].

Detailed historical records of a food product help to identify its links to a specific region and are one of the more important steps required in order to obtain European Union (EU) geographical indications, such as a Protected Designation of Origin (PDO) or protected geographical indications (PGIs).

Understanding the history of food products can also benefit the food industry. Entrepreneurs can draw inspiration from past techniques and ingredients to create new products that are unique and authentic. This can also help with marketing, where authenticity and tradition are often valued by consumers.

Given the current environmental issues, examining historical records can provide valuable information on sustainable agricultural and culinary practises used in the past. This approach can aid in creating future plans that are more environmentally friendly, yet efficient and adapted to local conditions.

This study presents the results of a search to find out the proof of the link between ”śląski” cheese and the Silesia region. Until now, no such studies have been conducted in Poland. Consequently, their distinctiveness is evident when evaluated on a national level. Silesian dairies, archives, and libraries were visited in search of the necessary documentation. This search was the first step towards a comprehensive treatment of the issue. Establishing an “origin link” between the cheese and Silesia would justify the possibility of taking action to protect the name “śląski” cheese (“Silesian” cheese) as a geographical indication. This was performed by finding archival documents, articles, and books on the link between cheese production and Silesia. As part of the research, a questionnaire was also carried out to briefly characterise consumers in terms of their behaviour on the regional cheese market. The research conducted can also serve as an example for those looking for a food product association that has been associated with a specific region over the years.

The article has been divided into three parts. The first of them explains how the materials have been obtained, which were the basis for further research. The second part presents in detail the results of the query conducted and the results of the survey. The last part refers to the European Union’s legal solutions for geographical indications—the Protected Designation of Origin (PDO) and protected geographical indication (PGI).

The main objective of the article is to prove that there is a strong “origin link” between “Silesian” cheese and the region Silesia. As a result, it may be possible to obtain protection for it as a geographical indication.

## 2. Materials and Methods

### 2.1. Query

A query is a systematic and purposeful research process to gather scientific information from a variety of sources. This search included Polish and international scientific databases (e.g., the National Digital Center—NDC, CEON Open Science Center Repository, Web of Science, Scopus), data library catalogues, journals and books, and a “Google Scholar” web search. A search for information on “śląski” cheese was carried out also in the following libraries: the National Library in Warsaw, the Silesian Library in Katowice, the Library of SGH Warsaw School of Economics, the Library of the Poznań University of Economics and Business, the Library of the University of Life Sciences in Poznań, and the Library of the Technical University in Łódź. Also, the following archives were used: the State Archive in Warsaw, The Central Archives of Modern Records in Warsaw, the State Archive in Katowice, the State Archive in Wrocław, and the State Archive in Gorzów.

Apart from the traditional paper archives, a large number of digitalised resources were examined. Sites of former cheese production in Katowice and Tychy were also explored.

Our query consisted of the following stages:

- establishing the subject of the study;
- selection of the relevant sources of information;
- selection of the key words;
- gathering the information;
- evaluation of the gathered scientific material and bibliographical sources;
- selection and recording of the information.

The query was a very “sharp” search, which means that only specific keywords were used. In our research “ser harceński” (“harceński cheese”) and Polish synonyms of this word, “kwargle” and “gomółki”, were used.

The query concerned one niche product that was produced in Poland before the Second World War, during the communist period (1945–1989), and for 4 years in independent Poland. The material was scattered and difficult to find. In the above-mentioned databases, entering the searched keywords in the NBC database resulted in three records being found, and in CEON, only one was found. For this reason, the work was based on library work and reading information about “śląski” cheese page by page in books and dairy journals without a time limit. The articles were then verified in terms of information about the place of production of the cheese. The rest were excluded. The basis for this article was information only about the production of “śląski” cheese and its place of production (Polish Silesia).

During the query, more than fifty documents related to “śląski” cheese were discovered, which clearly confirm the relationship of this cheese to the Silesian region. Over 156 titles were investigated, which included a number of annuals and periodic issues of journals.

The material collected relates to a type of ripened acid curd cheese, which in Poland, is called “harceński”, “domowy”, but also “kwargle” or “gomółka”. For the purposes of this article and due to the analysis of this cheese and its association with the Polish Silesia region, the unified working name “śląski” was adopted for it.

### 2.2. Survey

The aim of the survey was to investigate whether the consumers

- consciously choose products from a specific region,
- read labels on food products to check where the product was manufactured,
- buy cheeses and know their names,
- have encountered the names “Protected Designation of Origin, PDO” and “Protected Geographical Indication, PGI”, and
- identify their graphic symbols and where they have encountered them.

The current study on regional and traditional food was conducted on a group of Poles born in 2009 and earlier. The population size was n = 32,071,056 [7]. The minimum sample size (n = 267) was calculated using the sample calculator [8]. A total of 270 people took part in the survey. The significance level of the result was 95%, the estimation error was maximum 6%, and the percentage of the phenomenon in the general population was 50%. Given the selected study parameters, the group was quantitatively representative of the Polish population born up to and including 2009.

The questionnaire consisted of twelve closed-ended questions, four questions on population demographics, and eight on food. The demographic factors encompassed variables such as age, gender, educational attainment, and employment status. The questions were single-choice with the exception of one. The final question, “Where did you encounter the pictograms?”, was multiple choice and contained several answers because there was a possibility that the respondent could have more than one answer.

In compliance with the stipulations of Order No. 41/2023 of the Rector of the Poznań University of Life Sciences of 7 June 2023 on the Rector’s Committee for the Ethics of Scientific Research Involving Human Subjects, scientific research that uses anonymized human data that were collected for other purposes and anonymized before the researcher has access to it, and whose structure prevents any link to specific individuals is not subject to ethical review by the Rector’s Committee for the Ethics of Scientific Research Involving Human Subjects [9].

In addition, a pilot study (n = 20) was conducted to verify if the questions are understood. Prior to initiating the research, researchers adjusted the phrasing of certain questions and rearranged their sequence within the survey.

An online Google Forms survey related to food products was conducted between June 2024 and August 2024. Participation in the survey was voluntary. The survey questionnaire was shared through social media platforms, and the respondents who completed it were selected at random. Data were collected by means of the internet. Stratified sampling was not used.

The percentage results obtained (after the study was completed) were quoted directly in the article.

It should be explained that the authors decided to use Google Forms (knowing its advantages and disadvantages). They believe that in the case of this survey, the choice of Google Forms was appropriate and justified. The tool is simple and does not require any additional skills from the respondents. This resulted in a satisfactory number of responses. The questions were general and did not require an in-depth analysis of the respondents and their answers. It should also be emphasised that from the outset, the intention of the authors was not to use protected personal data. Accordingly, no personal data were collected to identify respondents. The authors believe that because of this, respondents were sincere and their answers were truthful. Of course, in any survey, it cannot be completely ruled out that respondents did not reveal their preferences. Finally, the collected responses allowed us to draw sufficient conclusions.

## 3. Results and Discussion

### 3.1. Ripened Acid Curd Cheeses

Ripened acid curd cheeses from skimmed curd are known and produced mainly in Germany (e.g., Handkäse, Harzer, Korbkäse), the Czech Republic (Olomoucké tvaružky), but also in Norway (Gammelost).

**Figure 1:**
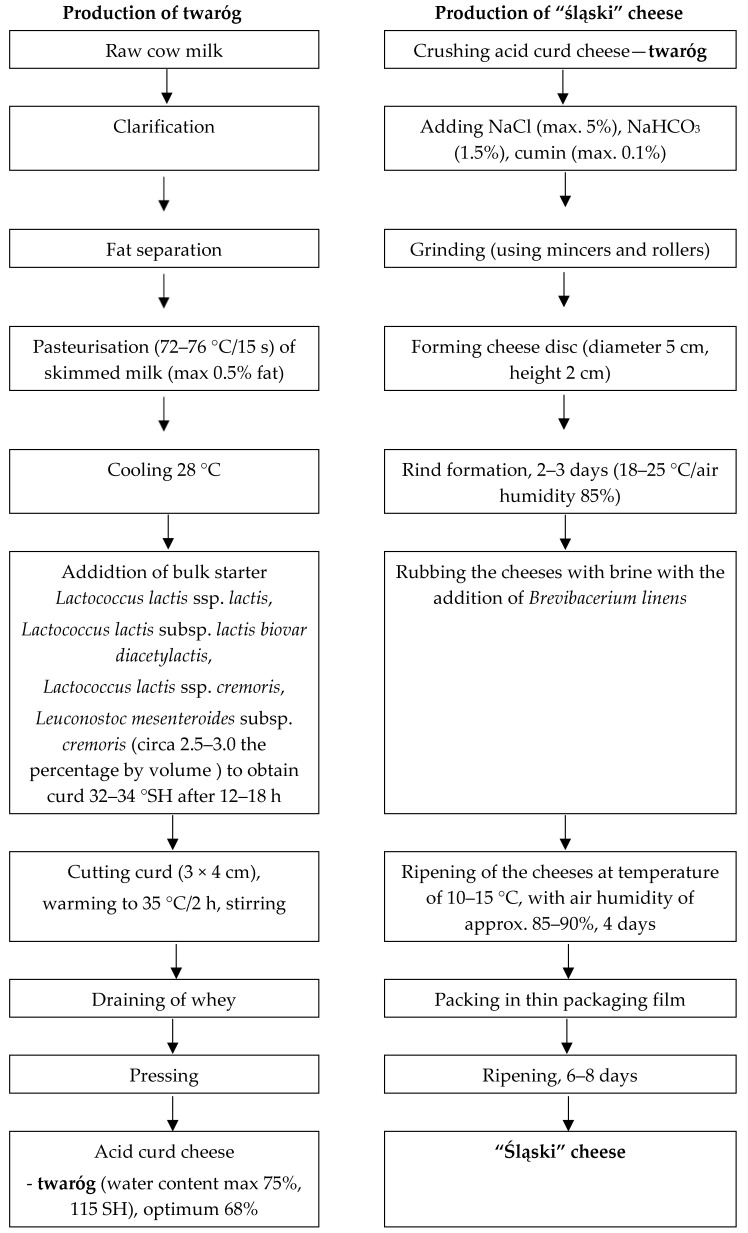
The production of twaróg and “śląski” cheese.

The production of ripening acid curd cheeses involves obtaining an acidic curd cheese (twaróg), adding other ingredients (ripening salt: CaCO_3_ and/or NaHCO_3_, salt, spices), and forming and ripening the cheeses (using brine and proteolitic microflora, e.g., Geotrichum *candidum*, *Brevibacerium linens*) [10,11]. In Poland, twaróg was produced in separate, independent plants. The cheese-making process had several steps. To emphasise the importance of each step, a flowchart is included in Figure 1.

The production method did not change until 1993, when its production continuity in Poland (in Silesia) was interrupted. “Śląski” cheese had to meet the following organoleptic and physicochemical requirements [12]:

Shape and dimensions—flat cylinder (diameter ca 5 cm, height ca 2–3 cm);

Rind—cream to yellow-brown;

Structure—soft, flexible;

Holes—none;

Colour—white to yellow;

Flavour—typical for this cheese, spicy, slightly sour;

Titratable acidity ˂ 80 °SH;

Moisture content ˂ 65%;

Salt content 2.5–3.5%;

Weight 40–63 g.

The whole manufacturing process of “śląski” cheese, from receipt of the purchased raw material to the packaging of the cheese, was always concentrated in the Silesia region (with Gliwice, Katowice, and Tychy as its historical, geographical, and economic centre). All stages of the manufacturing process practically ruled out any transport and/or handling of the unpacked cheese outside the manufacturing facility. The production documentation contained records of the suppliers of every batch of raw material, of the manufacturing operations, and of the purchasers of deliveries. Every package of the product was labelled with the producer’s name and address. This made it possible to trace the whole manufacturing process, which was under the permanent supervision of control bodies. The unique culture, environment, and place determine the uniqueness of regional cheeses, and this was also the case with the “śląski” cheese.

In 1932, the best-known ripened acid curd cheeses in central Europe were “Alte Kuhkäse”, “Berliner Kuhkäse”, “Märkische”, “Preβkäse”, “Rinnenkäse”, “Harz”, “Mainzer Handkäse”, and “Olomoucké tvaružky” [13,14].

Sometimes, the cheese names used were misleading because they were not derived from their place of production, such as the “Berliner Leichenfinger”, made mainly in Silesia in the Legnica area [13].

The interaction among Polish, German, and Czech cultures, combined with the consequences of warfare and shifts in borders, played a crucial role in influencing food production systems. An example of such interconnections is “Harzer” cheese (“harceński” cheese), which was produced not only in the Harz region (as its name might suggest), but also in the Polish part of Silesia.

### 3.2. Literature Evidence of the “Origin Link” Between “Śląski” Cheese and the Silesia Region

In Polish-language materials, the following names also appeared for “śląski” cheese: “harcki” [15],“hercyński” [16], “kwargle” [17,18], “harckie kwargle”, “gomółki harckie” [19], “domowy” [20], and “harceński”.

The oldest traces of the connection between the cheese and Silesia can be found in the daily newspaper the *Górnoślązak* for the date 5 April 1905 [21]. There is an advertisement for a seller of “harceński” cheese, who sold four pieces of “harceński” cheese at a price of 10 fenig. In the same newspaper, a piece of information appeared about a dishonest seller of “harceński” cheese, who sold this product at a higher price (9 fenig) than the maximum price set (7 fenig—per 40 g). According to a government regulation of 21 January 1915, a maximum price of 80 fen. per pound (453.6 g) was applicable to mature “harceński” cheese [22].

Advertisements for sellers of “harceński” cheese were placed in many Silesian newspapers, including the *Głos Śląski* [23], the *Dziennik Śląski* [24], the *Polak* [25], the *Gazeta Opolska* [26], and the *Katolik* [27].

The currencies with which cheese was sold changed. Initially, it was the mark, from 9 December 1916, it was the Polish mark, and finally—as a result of the monetary reform of 29 April 1924—the Polish mark was replaced by the Polish zloty.

**Figure 2:**
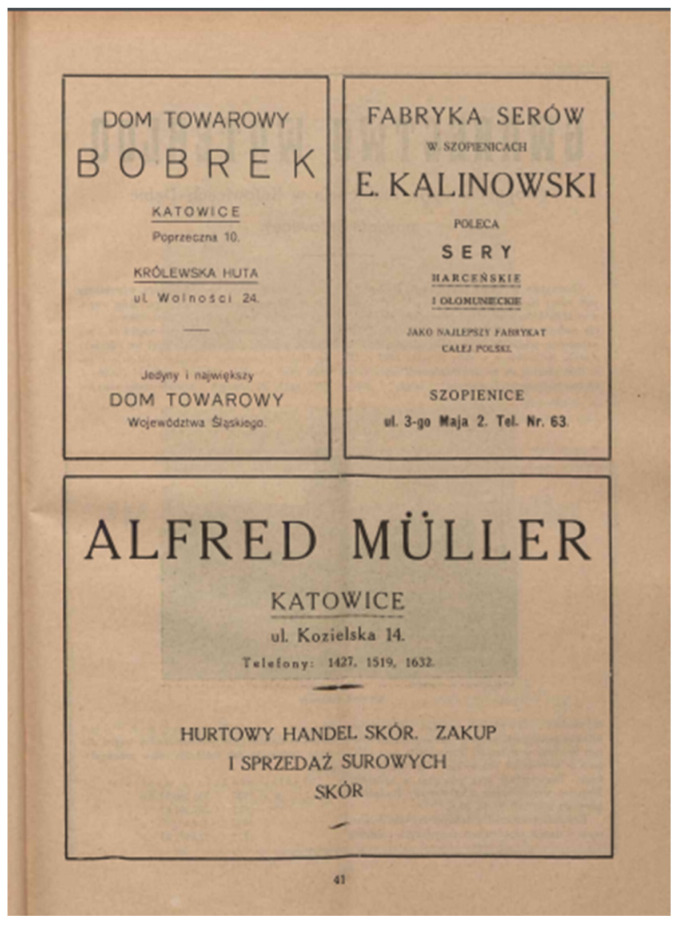
Advertisement of the cheese factory E. Kalinowski (top right corner) placed in the Silesian issue of the monthly magazine “Morze” (public domain) [29].

The earliest documented mention of “śląski” cheese manufacturing in Silesia originates from the year 1923. Kalinowski placed an advertisement for his factory in the programme of the Polish Theatre in Silesia [28]. Just how industrious the owner of this factory was is evidenced by advertisements that can be found in many magazines. The advertisement shown in Figure 2 was placed in the Silesian issue of the monthly magazine “Morze” in 1927 [29].

**Figure 3:**
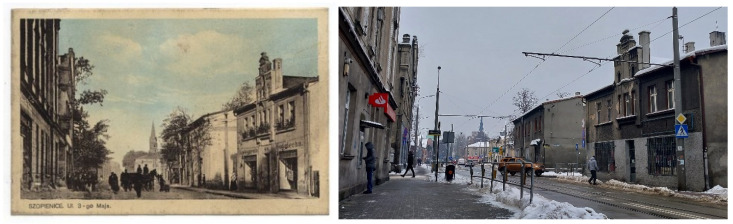
Szopienice [name of the city in Poland] in the past (public domain) [30] and present (Photo S. Chudy).

In the collection at the Silesian Digital Library, there is a postcard (Figure 3) from Szopienice dated 1922–1926 [30]. It depicts 3rd May Street, with the Kalinowski dairy building on the left side. The layout of the buildings shown in the postcard remains unchanged to this day; however, the street name has been updated to Wiosny Ludów Street.

Even before the Second World War, the Silesian cheese factory passed into the hands of Julian Kalinowski. The book published in 1938 contains an advertisement for the maker of “harceński” cheese, Julian Kalinowski [31]. After the Second World War, private factories in Poland (including dairies) were closed down or taken away for the benefit of the state. The last advertisement for the factory that the authors found was dated 22 July 1948, from the magazine “Naprzód” [32]. After 1948, the authors no longer found advertisements for this dairy. Today, the building of the former dairy is used for housing purposes and is inhabited by descendants of J. Kalinowski.

**Figure 4:**
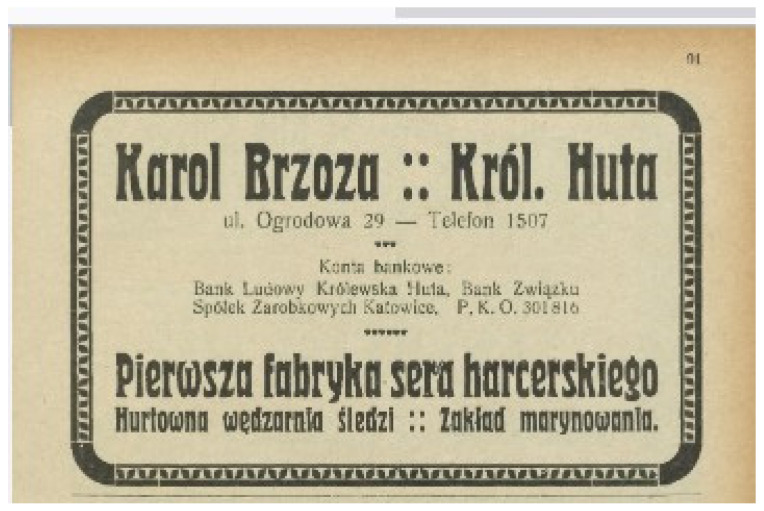
Advertisement of “harcerski” cheese from the “Address book of industry, trade and finance of Polish Upper Silesia 1924/25” (public domain) [33].

In 1924, K. Brzoza placed an advertisement for his cheese (Figure 4) in the “Address Book of Industry, Trade and Finance of Polish Upper Silesia 1924/25” [33]. It is noteworthy that the advertisement uses the name “harcerski” cheese instead of “harceński” (one letter difference in the name). This may be a mistake, but it is more likely to be a deliberate act by an entrepreneur who wanted to open the first factory of this cheese. The use of the term the first factory of “harceński” cheese would have been untrue, hence the change in name from “harceński” to “harcerski” cheese.

Additionally, six plants where “harceński” cheese was produced before Second World War were found: Szarlociniec [34,35], the Silesian Dairy Plant [36], and three dairies located in the Wrocław Voivodship [37,38,39]. In the area of the city of Gliwice, the sixth and the last pre-war dairy was found. After the war, it was heavily damaged, with machinery and equipment being taken away [40,41]. Soviet troops who entered Gliwice in January, 1945, “liberating” the city, within 2 months, burned 30% of the buildings, dismantled most of the plant equipment, and took it eastwards to the USSR—Union of Soviet Socialist Republics. After the Second World War, production of “harceński” cheese was quickly resumed in Silesia [42].

In order to standardise product characteristics, between 1951 and 1955, the Minister for the Meat and Dairy Industry established a set of Ministry quality standards, including the RN-A/Ml-51/43 standard for “harceński” cheese [43]. In 1962, this was replaced by the standard PN-62/A-86246 ripened acid curd “harceński” cheese [12].

The newspaper *Trybuna Robotnicza* on the 8 November 1955 reported a since-forgotten fact in Poland: there were two “harceński” cheese factories in the Silesia region after the Second World War. In 1955, curd was delivered to these factories in barrels, then placed in large vats where it was mixed with salt, caraway, and baking soda. This mass was ground to a paste-like consistency and fed into the moulding machine. After maturing, cheese was packaged and sold [44]. “Harceński” cheese was a local product in Poland, being of importance in the Silesia region [45]. The tradition of consumption of “harceński” cheese influenced the location of its production plants in Poland [46,47].

In 1954, the entire Polish dairy assortment consisted of just 28 products, including “harceński” cheese [48]. Production increased from year to year, and in 1955, it was 385,000 kg, in 1960—760,000 kg, in 1965—981,000 kg, in 1966—1,001,000 kg, in 1967—1,146,000 kg, and in 1968—1,050,000 kg [49]. It was also an export product, and the only producer of it (for export) was precisely the Gliwice plant [50]. The export volume in the years 1960–1961 in the period 1.01–31.08 was, respectively, 121,000 and 195,000 kg [51]. The export price of “harceński” cheese was 21 zł per 1 kg [52].

The dairy plant in Gliwice had a production capacity of 1,800,000 kg/year. The plant had the following machinery and equipment: two curd mincers, three gravity rollers, and three moulders for “harceński” cheese [53].

**Figure 5:**
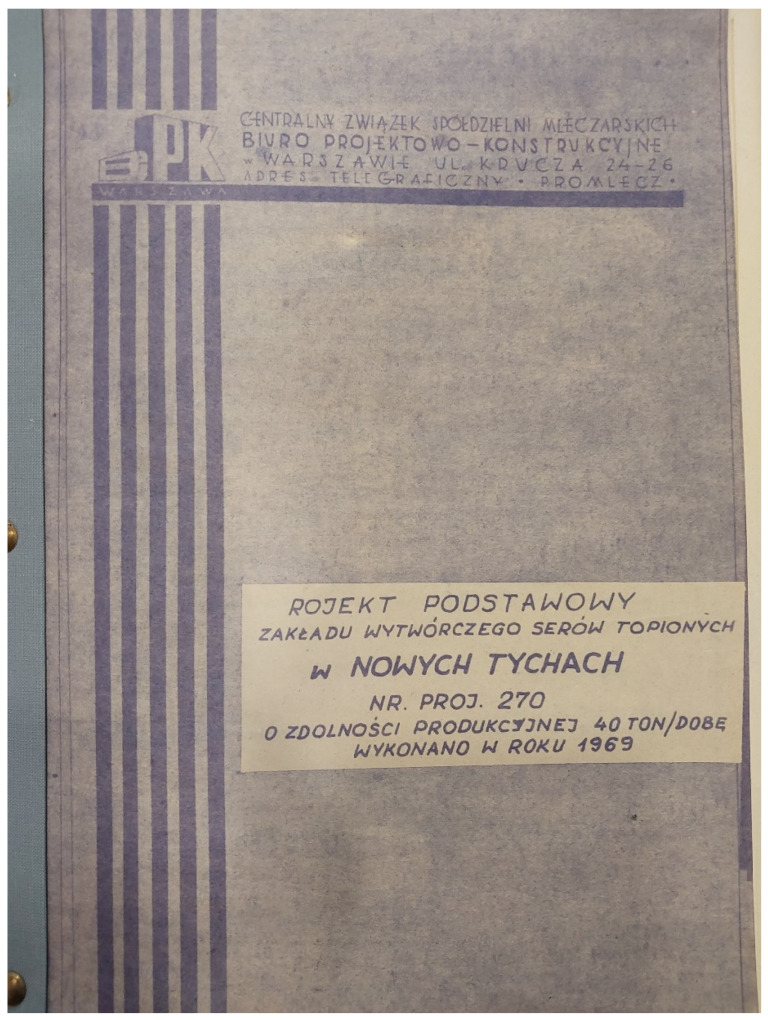
Cover of the design of the Tychy [name of the Polish city] processed cheese and “harceński” cheese factory in 1969 (Photo S. Chudy).

As early as the 1960s, there were plans to build a new melted cheese and “harceński” cheese factory in Tychy. In the newspaper *Trybuna Robotnicza* in 1973, there was an article about a cheese factory under construction in Tychy [20]. It stated that the planned range of products would also include “harceński” cheese. To ensure the freshness of this short-shelf life product, it was to be “released” in an immature state. A blueprint of the cheese factory from 1969 has survived to this day. Figure 5 shows its cover. In the title, “The basic project of the New Tychy Melted Cheese Manufacturing Plant. Project Number 270 with a production capacity of 40 tons per day, prepared in 1969”, there is no information about “harceński” cheese, but the documentation includes drawings of the cheese production hall, the layout of the equipment, and the work schedule of the machines. The plant was opened on 11 November 1973, and production plans for 1975 called for 1,500,000 kg of “harceński’ cheese [54].

“Harceński” cheese was seen as an excellent snack to accompany beer; it was a high protein, dietary product because it was almost fat-free. The starting material was milk protein in the form of skim acid curd, which was subjected to special maturation processes involving bacterial cultures. In 1974, the growing production of “harceński” cheese was seen as “preserving” a huge amount of valuable protein through being processed into casein, which, among other things, was used for industrial purposes (e.g., to make buttons) [55].

In 1976, the Tychy plant produced 469,423 kg of “harceński” cheese. The report on the Polish dairy plants’ operations between 1975 and 77 shows that no attempt was made to expand “harceński” cheese production on a nationwide scale, and it remained a local speciality [56].

The most recent reference to “harceński” cheese appeared in a 1993 advertisement published in the Polish dairy journal. The picture shows the president of the dairy with the product range (including “harceński” cheese) of SERTOP Tychy [57].

At the beginning of the 1990s, the profitability of milk production in Poland dropped significantly. The decline in milk production was accompanied by a massive influx of imported dairy products, often of much lower quality than domestic products and sold at lower dumping prices (usually products subsidised under EU interventionism). In 1994, about 700 dairy plants belonging to about 320 cooperatives and about 120 private companies were involved in milk processing. At that time, private dairy companies occupied around 20% of the market. In 2002—with a similar number of private establishments—they already occupied almost half of the dairy market [58].

In the 1990s, the Silesian “harceński” cheese produced by SERTOP Tychy disappeared from the Polish market.

### 3.3. The Polish Consumer on the Regional Food Market

Two hundred and seventy people took part in a survey on Polish consumer knowledge of regional food. The largest group of respondents (48%) were the youngest, born between 1997 and 2009 (aged 15–27 years); 8% of respondents were born up to 1964 (˃60 years); 32% between 1965 and 1980 (44–59 years); and 12% between 1981 and 1996 (28–43 years). The age ranges corresponded to the boomer generation (older and born up to 1964 and generation X, Y, and Z [59].

Additionally, 64% of the respondents were female, 35% were male, 0.5% did not answer the question about gender, and 0.5% marked “other”. The majority of those who took part in the survey declared a university or secondary education, respectively, at 57% and 40%. Primary education was declared by 2% and vocational education by 1% of the respondents.

The majority of respondents (42%) worked in the service sector (trade, transport, communications, utilities, health care, education, and tourism and culture) or studied (38%), 10% worked in the industrial sector (manufacturing and construction), 7% were pensioners or did not work, 2% of respondents worked in the agricultural sector (agriculture, forestry, and mining), and 1% were primary and secondary school students.

In addition to the statistical section, the survey contained eight questions related to consumer knowledge and behaviour in the regional food market (Table 1).

**Figure 6:**
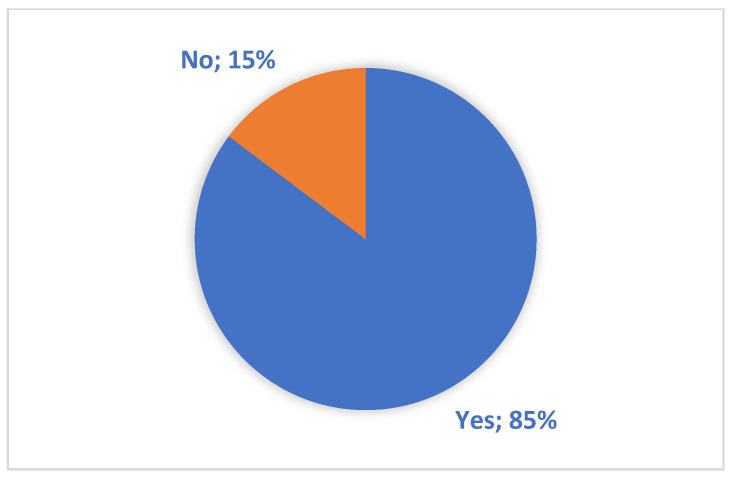
Respondents’ answers to the question when shopping, do you happen to choose products from a particular place, e.g., country or region?

The answers to the first food-related question show that 85% of the respondents are consumers who are open to global and regional offers and consciously choose products from a specific region (Figure 6).

Question two, “Do you pay attention to pictograms on food labels?”, was aimed at verifying whether the efforts of producers trying to convey certain information in the form of drawings, including, among others, information on Protected Designation of Origin (PDO) and protected geographical indication (PGI), are justified. Respectively, 15% and 70% of participants responded, claiming that they always and sometimes pay attention to the pictograms. The message conveyed by pictograms did not reach 15% of respondents.

The third question, which was intended to check the reliability of the answers given in question 1, was “Do you read labels to check where food is produced?” The results, “always”—17%, “sometimes”—69%, and “never”—14%, confirm the cohesion of answers obtained from first and third questions.

**Figure 7:**
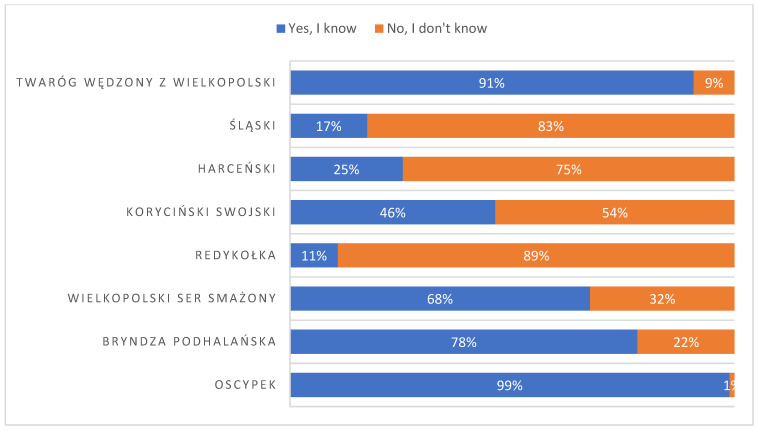
Answers to the question “Do you know the cheeses listed?”.

The following question examined the awareness of regional cheese among the respondents. The survey shows that respondents know only some types of regional cheeses (Figure 7). The survey listed five Polish regional cheeses with the EU logo (“koryciński swojski”, “redykołka”, “wielkopolski ser smażony”, “bryndza podhalańska”, and “oscypek”). Additionally, “twaróg wędzony z Wielkopolski” cheese, “harceński” cheese, and “śląski” cheese appeared in the survey. The most well-known cheeses (above 50% recognition) were “oscypek”, “twaróg wędzony z Wielkopolski”, “bryndza podhalańska”, and “wielkopolski ser smażony”.

In contrast to earlier studies that also examined regional cheeses from Poland, the recognition levels for these cheeses were as follows: “oscypek” remained constant, while “koryciński swojski” saw a 14% increase and “bryndza podhalańska” experienced a 5% rise. A decrease in knowledge about the name of the cheese was noted for “redykołka” and “wielkopolski ser smażony” by 8% and 3%, respectively [60].

This article focuses on the examination of ripened acid curd cheese originating from Silesia, which has been known by various names throughout history. Therefore, the term “śląski” cheese, translating to “Silesian” cheese, has been selected as the working designation for this product. Unfortunately, 17% of respondents declared that they were familiar with the name “śląski” cheese, although no cheese with this name was commercially available.

To test shopping preferences for cheese, the question “Do you buy these (listed in the survey) cheeses?” was asked. The regional cheese most frequently bought (and therefore most liked) by respondents was “oscypek”. The answer “yes” was marked by 76% of respondents. Only one other cheese was characterised by high (55%) purchase interest among respondents—“twaróg wędzony z Wielkopolski”. The other cheeses, “wielkopolski ser smażony”, “koryciński swojski”, “bryndza podhalańska”, “harceński”, and “redykołka”, were bought by, respectively, 39%, 26%, 25%, 10%, and 4% of respondents.

Such low interest in “redykołka” cheese may be explained by the fact that the word “redykołka” cheese is used for a small (30–60 g) version of “oscypek” cheese (600–800 g) mainly in regional dialect. It has not been yet acknowledged in consumers’ consciousness. Most Poles use only one name, “oscypek”, for both cheeses. It should be noted that there is only a little difference in the technology used between “oscypek” cheese and its smaller counterpart, “redykołka” cheese.

The final three questions related to EU pictograms, which are EU designations for regional foods.

Less than half of the respondents (45%) answered that they had encountered the name “Protected Designation of Origin, PDO”, 43% of respondents answered that they had not encountered the name, and 12% did not remember encountering the name at all. Even fewer had encountered the name “Protected Geographical Indication, PGI”. Only 36% declared that they had encountered this name, 52% believed that they had not encountered this name, and 12% did not remember. The results for “Traditional Speciality Guaranteed TSG” name recognition were, respectively, 44% (“yes”), 45% (“no”), and 11% (“I do not remember”).

**Figure 8:**
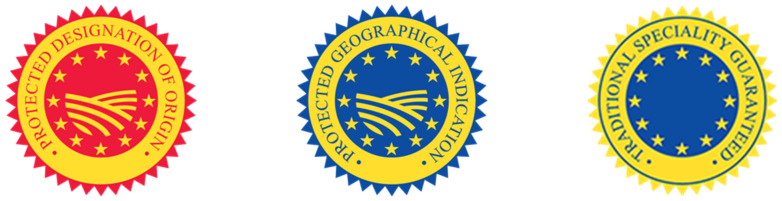
Labels of Protected Designation of Origin, protected geographical indication, and Traditional Speciality Guaranteed products [62].

When asked do you recognise the following pictograms (Figure 8)?, 57% of the respondents marked the answer “yes”; the remainder marked the answer “no”. Compared to previous studies by Chudy and Gierałtowska, a significant improvement in the recognition of these pictograms was observed [61]. In studies conducted in the years 2010–2012, 1% of respondents claimed that they had seen the EU logos (PDO, PGI, or TSG)

The last question (the only multiple-choice) asked where respondents encountered the above-mentioned signs. Most respondents encountered the symbols in traditional sales (shop or stall) with 52%, on the Internet—15%, on TV—14%, during direct sales (e.g., at shows)—12%, and at a friend’s house—3%. Furthermore, 37% of respondents stated that they had not seen these pictograms anywhere.

## 4. The Role of Geographical Indications (GIs)

Currently, Poland is a leading cheese producer and ranks fifth in the European Union and seventh in the world. The largest producers (in million tons) of cheese are United States, 6.4; Germany, 2.6; France, 1.9; Italy, 1.3; Russia, 1.1; Netherlands, 1.0; and Poland, 1.0 [63,64].

The structure of Polish cheese production is dominated by cottage cheese (51% share) and rennet matured cheeses (37%). Of lesser importance are processed cheeses, 8%, and other cheeses, 4%. Poland’s exports of cheese products increased as much as fourfold between 2004 and 2020 [65].

It is undisputed that “indications of the geographical origin of goods (…) regularly provide valuable input into decision-making processes. This applies in particular where the provenance of the product has a direct influence on the objective quality—the taste and other properties—of the varieties” [66]. Thus, the geographical indications can play a special role in promoting “ślaski” cheeses originating from a specific region—Silesia. The concept of geographical indications is based on the so-called “origin link”, which is a “set of rules that identify the elements whose presence must be proved in order to establish a connection between a product and a place. If, on the basis of these rules, such a connection is found to exist, the name that identifies the origin of the good, usually of a geographical location, is granted legal protection under Intellectual Property Law” [67]. It must be considered whether, at this stage of research, an “origin link” between the cheese and Silesia is proven. Such indications create associations in the minds of consumers between a particular good and a particular geographical area (territory, region, city, etc.).

The aim of safeguarding product names through geographical indications is to foster variety and highlight items that possess unique traits associated with their place of origin. They also ensure that the consumer is not misled when choosing a product and is guided by clear and concise information on its origin. Indeed, the consumer associates certain specific characteristics of a product with a certain geographical area that distinguishes it from goods of the same type. The system of protected geographical indications can also influence consumer decision-making.

However, the protection of geographical indications is intended not only to protect the consumer but also the producers of the same type of product, who loyally refrain from using certain indications if they do not correspond to the actual state of affairs [68]. The protection granted also ensures that the reputation of products originating from a given geographical area is not undermined by other products of the same type. Studies also show that geographical indications accelerate innovation and development [69]. GIs are also a valuable tool to use in an attempt to foster local development processes and to increase agricultural economic sustainability [70]. It has also been stated in the preamble to the Regulation (EU) 2024/1143 of the European Parliament and of the Council of 11 April 2024 on geographical indications for wine, spirit drinks and agricultural products, as well as traditional specialities guaranteed and optional quality terms for agricultural products, amending Regulations (EU) No 1308/2013, (EU) 2019/787, and (EU) 2019/1753 and repealing Regulation (EU) No 1151/2012 (Regulation 2024/1143) that “the use of geographical indications rewards producers fairly for their efforts in producing a diverse range of quality products. At the same time, that can benefit the rural economy, which is particularly the case in areas with natural or other specific constraints, such as mountain areas and remote regions, including the outermost regions, where the farming sector accounts for a significant part of the economy and production costs are high. In that way, quality schemes are able to contribute to and complement rural development policy as well as market and income support policies of the common agricultural policy” [71].

Thus, it is emphasised in the literature that the role of geographical indications cannot be overestimated, and it has therefore become necessary to give them a specific legal framework [72]. Currently, the system of protection of geographical indications for agricultural products in the EU is regulated in Regulation 2024/1143. The regulation established a single and complete system of geographical indications, protecting the names of wines, spirits, and agricultural products with characteristics, qualities, or reputations linked to their place of production. This system is based on the registration of geographical indications in the EU register [62].

Geographical indications for agricultural products are referred to as a “protected designation of origin” or “protected geographical indication”. Under Article 46 of the Regulation, a “designation of origin” for an agricultural product is a name which designates a product originating in a specific place, region, or, in exceptional cases, country; the quality or characteristics of which are essentially, or exclusively, due to a particular geographical environment comprising natural and human factors; and all stages of whose production take place in the defined geographical area. Examples of such Polish cheese names are “redykołka” (PDO-PL-0588), “oscypek” (PDO-PL-0451), and “bryndza podhalańska” (PDO-PL-0450).

A “geographical indication”, on the other hand, is a name used to designate a product that originates from a specific place, region or country; whose specific quality, reputation or other characteristic is mainly due to this geographical origin; and of which at least one stage of production takes place in the defined geographical area. Poland has registered the following cheese names as geographical indications: “koryciński swojski” cheese (PGI-PL-0835) and “wielkopolski ser smażony” (PGI-PL-0551).

European Union logos for GIs have also been established to label and publicise the geographical indications. They may only be used for food produced by the corresponding product specification. They may also be used for information and educational purposes, provided that such use does not mislead the consumer.

At the same time, it is important to be aware that, compared to other leading cheese producers in the EU (Germany, Italy, and France), Poland has few cheese names protected as geographical indications, either as a PDO and PGI.

The EU register of agricultural product names “eAmbrosia” (as of 16 September 2024) shows that Poland has thirty-six registered protected designations of origin and protected geographical indications for food, including five for cheeses [62]. Additionally, “twaróg wędzony z Wielkopolski” has the Traditional Speciality Guaranteed (TSG) designation, which is associated with tradition. Germany has a total of 97 registrations, including 9 for cheeses; Italy has a total of 324, including 56 for cheeses; and France has 272, including 56 for cheeses. Poland, therefore, despite being the EU’s fifth largest cheese producer, has the fewest such registrations of the largest cheese producers.

## 5. Conclusions

A. The historical documentation of food manufacturing is an important tool for understanding the past and also a valuable source of knowledge. Preserving this information protects cultural heritage and supports education and research. It also helps to create sustainable and innovative solutions in the food industry. In the face of dynamic social, technological, and ecological changes, these records become an invaluable resource to help connect the past with the present and the future.

B. From the research carried out, it emerged that ripened, non-fat acid curd cheese made from skimmed milk had been produced in Silesia since at least 1923. It also succeeded in demonstrating the origin link between cheese and Silesia. Thus, this type of cheese could be called “śląski” cheese.

C. However, at present, the cheese is not produced nor is it protected in any way. Restoring production and obtaining protection for “śląski” cheese would be important for Poland as a significant cheese producer in the EU and worldwide. It may also have an impact promoting the Silesia region.

D. The survey results proved that Polish consumers consciously choose products from a particular place or region. Most of them familiarise themselves with labels and indications on packaging before making a purchase.

E. Although less than half of those surveyed are familiar with the names “Protected Designation of Origin” and “Protected Geographical Indication”, most recognise their graphic symbols. Furthermore, the vast majority of respondents know the names of Polish cheeses whose names are protected, in particular “oscypek”, “bryndza podhalańska”, and “wielkopolski ser smażony”. On this basis, it can be assumed that obtaining protection for “śląski” cheese (“Silesian” cheese) in the form of a PDO or PGI could have a significant impact on consumer choices.

F. Further research should be carried out to determine whether it would be possible to obtain a protection for geographical indications for other cheeses produced in other regions of the Poland.

## Figures and Tables

**Table 1:** Questions included in the survey.

No	Question
1	When shopping, do you happen to choose products from a particular place, e.g., country or region?
2	Do you pay attention to pictograms on food labels?
3	Do you read labels to check where food is produced?
4	Do you know the cheeses listed?
5	Do you buy these (listed in the survey) cheeses?
6	Did you encounter these names?(“Protected Designation of Origin, PDO”, “Protected Geographical Indication, PGI”, “Traditional Speciality Guaranteed”)
7	Do you recognise the following pictograms?
8	Where did you encounter the pictograms?

## Data Availability

The original contributions presented in the study are included in the article; further inquiries can be directed to the corresponding author.
